# Therapeutic Approaches to Neuroprotective Activity by Complementary and Alternative Medicines

**DOI:** 10.1155/2012/376068

**Published:** 2012-10-17

**Authors:** Ilkay Erdogan Orhan, Monica Rosa Loizzo, Mahmud Tareq Hassan Khan

**Affiliations:** ^1^Department of Pharmacognosy, Faculty of Pharmacy, Gazi University, Ankara, Turkey; ^2^Department of Pharmacognosy and Pharmaceutical Botany, Faculty of Pharmacy, Eastern Mediterranean University, Gazimagusa, Famagusta, The Northern Cyprus, Turkey; ^3^Department of Pharmaceutical Sciences, Faculty of Pharmacy, University of Calabria, Rende, Italy; ^4^Center for Pharmaceutical Biotechnology, College of Pharmacy, University of Illinois at Chicago, Chicago, IL 60607-7173, USA

Neurodegeneration is a large-capacity term which can be described as progressive damage on neurons in brief. Many diseases are known to be associated with central nervous system such as Alzheimer's disease (AD), Parkinson's disease (PD), Huntington's disease (HD), multiple sclerosis (MS), and amyotrophic lateral sclerosis (ALS/Lou Gehrig's Disease) and occur as a result of neurodegenerative processes. Actually, the neurodegenerative diseases have a quite higher prevalence among the elder population living especially in the well-developed countries because of the higher life standards. For instance, AD is estimated to affect approximately 5 million people only in the USA. 

Although pathogeneses of most of the neurodegenerative diseases are still continuous topics of a common research all over the world, the principal mechanisms linked to these diseases have been frequently suggested as genetic mutations and intracellular mechanisms in specific brain regions such as protein degradation pathways, misfolding or occurrence of abnormal protein structure. Consequently, neuroprotection is an important treatment option for such neurodegenerative disorders. The extensive research on discovery novel drug candidates has shown that natural products such as plant extracts and plant-originated compounds have enormous potential to become drug leads with neuroprotective activity. One of the most impressing examples of those drugs is galanthamine, an alkaloid isolated from the bulbs of the snowdrop plant (*Galanthus* sp.) that has become a licensed and approved medicine as cholinesterase inhibitor for the treatment of AD. 

Considering the widespread research on the role of herbal medicines in neuroprotection, 13 research or review articles that contained interesting results relevant to the subject have been included in the current special issue. A particular emphasis has been given to the articles dealing with neuroprotective effect of eastern herbal medicines used in China, Korea, Taiwan, Malaysia, and India. Among the papers, Y. Piao and X. Liang contributed with an excellent review about neurological activity of traditional Chinese medicine against diabetic peripheral neuropathy (DPN) associated with chronic diabetes mellitus that causes nerve degeneration while in another review article I. E. Orhan summarized the literature relevant to neurobiological effects of *Centella asiatica *“gotu kola,” a reputed plant used as brain tonic in Ayurvedic medicine. 

G. Chan et al. investigated neuroprotective effect of the phenylcoumarin-type of compounds isolated from the Malaysian plant *Mesua kunstleri* using neuronal cell culture techniques, which led to identification of mesuagenin C as the promising neuroprotective agent. 

H.-C. Chang et al. reported *in vivo* neuroprotective effect of a Chinese formulation called Wei Na Kang (WNK), mainly consisting of *Panax ginseng*, *Ginkgo biloba*, and *Crocus sativus *using APP/PS1 transgenic mice and concluded that WNK has been found to reduce the decline in spatial cognition, which might be due to its effects on reducing A**β** plaque formation and ameliorating histopathology and ultrastructure in hippocampus of APP/PS1 mouse brain. Another study by Y.-L. Wang et al. brightened strong *in vivo *anxiolytic effect of the compound zhi zhu xiang (CZZX) originated from a clinical experiment that was reported to be an effective and well-tolerated antianxiety prescription in China. The findings by these authors pointed out to the statement that the anxiolytic efficacy of CZZX may be mediated by benzodiazepine binding site modulation at *γ*-aminobutyric acid-A receptors. A.-H. Yao et al. also contributed with an article that aimed to examine the role of toll-like receptors (TLRs) myeloid differentiation factor 88- (MyD88-) dependent pathway in the spinal cord injury (SCI) in adult male rats. They showed that MyD88 inhibitory peptide (MIP) by intramedullary application caused a significant improvement in recovery of locomotor function. On the other hand, rhynchophylline (Rhy), a tetracyclic oxindole alkaloid and the active component isolated from *Uncaria* species used for neurological conditions in traditional Chinese medicine, was investigated by D. D. Xu et al. against methamphetamine (MA) neurotoxicity using neuronal cell culture system and demonstrated to exert inhibitory effect against MA impairment under *in vitro* conditions. S.-Y. Su et al. revealed protective role on memory after ischemic stroke of paeonol, the simple phenolic isolated from the Chinese herb *Paeonia suffruticosa via* reducing amyloid precursor protein (APP), beta-site APP-cleaving enzyme (BACE), and apoptosis. Y.-C. Lo et al. investigated neuroprotective effect of San-Huang-Xie-Xin-Tang (SHXT), another traditional Chinese medicine containing *Coptidis rhizoma, Scutellariae radix*, and *Rhei rhizoma *against PD using the 1-methyl-4-phenylpyridinium (MPP^+^)/1-methyl-4-phenyl-1,2,3,6-tetrahydropyridine (MPTP) models. By all parameters used in this study, the authors suggested that SHXT exerted promising protection against MPTP-induced neurotoxicity in PD through its antioxidative and antiapoptotic effects. In C. Lv et al.'s study, quercetin, the most studied bioflavonoid up to date, was explicated against MPTP neurotoxicity related to PD using the similar models to Y.-C. Lo et al.'s work. C. Lv et al. revealed that quercetin treatment evidently improved the motor balance and coordination of MPTP-treated mice, which led to the comment that quercetin has antiparkinsonian properties.

In another paper, Y.-Y. Sunwoo et al. evaluated neuroprotective role of gongjin-dan, a Korean herbal mixture that contains *Moschus moschiferus*, *Corni fructus*, *Angelica gigantis radix*, and *Cervi parvum cornu* against transient middle cerebral artery occlusion (MCAo) connected with ischemic stroke using adult male Sprague-Dawley rats to constitute cerebral ischemic model. According to their results, gongjin-dan gained the advantage over the control groups in behavioral and immunohistological tests employed. Neuroprotective action of another Korean herbal formulation “Sibjeondaebo-tang” (SJDBT), which has been used to treat neuropsychiatric disabilities in traditional Korean medicine, has been explicated by H. S. Lim et al. against A**β** peptide-induced damage using *in vitro* culture and *in vivo* rat brain systems and elucidated to possess a protective role from neuronal damage in the experimental systems used. N. Li et al. reported antioxidant and anti-inflammatory activity of NNMBS275, consisting of the ethanol extract of *Viola patrinii* from Korea in in murine hippocampal HT22 cells and BV2 microglia. These authors stated that the neuroprotective and anti-inflammatory effects of NNMBS275 were linked to the upregulation of nuclear transcription factor-E2-related factor 2-dependent expression of heme oxygenase-1 in HT22 and BV2 cells.

The aforementioned papers published in this special issue represent quite attractive and exciting results about a number of plant extracts and herbal compounds in neuroprotection. In most of these papers, the mechanisms underlying their neuroprotective properties have been also explained using numerous experimental parameters. As the guest editorial team, we would like to express our deep appreciation to the authors of these articles, our reviewers, and the Editor-in-Chief and Editorial Assistants of ECAM who helped us to make this issue possible to publish.

*Ilkay Erdogan Orhan* *Ilkay Erdogan Orhan* *Monica Rosa Loizzo* *Monica Rosa Loizzo* *Mahmud Tareq Hassan Khan* *Mahmud Tareq Hassan Khan*

